# Metformin Targets the Metabolic Achilles Heel of Human Pancreatic Cancer Stem Cells

**DOI:** 10.1371/journal.pone.0076518

**Published:** 2013-10-18

**Authors:** Enza Lonardo, Michele Cioffi, Patricia Sancho, Yolanda Sanchez-Ripoll, Sara Maria Trabulo, Jorge Dorado, Anamaria Balic, Manuel Hidalgo, Christopher Heeschen

**Affiliations:** 1 Stem Cells & Cancer Group, Molecular Pathology Programme, Spanish National Cancer Research Centre (CNIO), Madrid, Spain; 2 Gastrointestinal Cancer Clinical Research Unit, Clinical Research Programme (CNIO), Spanish National Cancer Research Centre, Madrid, Spain; Cleveland Clinic, United States of America

## Abstract

Pancreatic ductal adenocarcinomas contain a subset of exclusively tumorigenic cancer stem cells (CSCs), which are capable of repopulating the entire heterogeneous cancer cell populations and are highly resistant to standard chemotherapy. Here we demonstrate that metformin selectively ablated pancreatic CSCs as evidenced by diminished expression of pluripotency-associated genes and CSC-associated surface markers. Subsequently, the ability of metformin-treated CSCs to clonally expand *in vitro* was irreversibly abrogated by inducing apoptosis. In contrast, non-CSCs preferentially responded by cell cycle arrest, but were not eliminated by metformin treatment. Mechanistically, metformin increased reactive oxygen species production in CSC and reduced their mitochondrial transmembrane potential. The subsequent induction of lethal energy crisis in CSCs was independent of AMPK/mTOR. Finally, in primary cancer tissue xenograft models metformin effectively reduced tumor burden and prevented disease progression; if combined with a stroma-targeting smoothened inhibitor for enhanced tissue penetration, while gemcitabine actually appeared dispensable.

## Introduction

Pancreatic ductal adenocarcinoma (PDAC) remains one of the most devastating cancers, and is the fourth leading cause of cancer-related deaths in industrial countries with a 5-year survival rate of less than 5% [1]. Many risk factors including smoking, alcohol consumption, and chronic pancreatitis have been recognized as potential risk factors for the development of PDAC [2]. Epidemiologic studies also suggest that diabetes mellitus, particularly type 2, is associated with enhanced risk for PDAC [3], [4]. Therefore, investigators have embarked on finding a putative link between the use of anti-diabetic drugs and a reduced risk for the development and/or progression of PDAC. Strikingly, in a retrospective analysis, oral administration of metformin in patients with diabetes mellitus type II was found to be associated with reduced risk for developing PDAC [5] as well as better outcome in patients with established PDAC [6].

The primary systemic effect of metformin (Met) represents a decrease in blood glucose levels via reduced hepatic gluconeogenesis and increased glucose uptake in peripheral tissues [7]. Mechanistically, metformin indirectly activates AMP-activated protein kinase (AMPK) signaling [8] and subsequently inhibits mTOR activity, which is frequently increased in cancer cells [9] including pancreatic cancer stem cells (CSCs) as a highly tumorigenic subpopulation [10]. This inhibitory effect of metformin on AMPK/mTOR signaling results in reduced protein synthesis and cell proliferation [11], [12]. Moreover, in established PDAC cell lines metformin is also capable of inhibiting PDAC [13]. Intriguingly, another recent study suggested that CSCs could be targeted by metformin via re-expression of miRNAs implicated in differentiation, although these data are based on non-validated cancer cell line-derived CSCs [14].

Unlike the majority of differentiated cells within the tumor, CSCs have been shown to be highly resistant to chemotherapy [15]. Therefore, drugs that selectively target CSCs may represent a more effective approach to overcome resistance and/or treatment relapse in PDAC. Here, we now provide compelling evidence that CSCs derived from a diverse set of primary human PDACs are highly vulnerable to metabolic reprogramming by metformin resulting in long-term survival of preclinical mouse models.

## Results

We have previously shown that primary pancreatic CSCs can be enriched *in vitro* as anchorage-independent three-dimensional spheres, which are enriched for cells with stem cell-like properties [15]. A total number of nine human PDAC xenografts were used with A6L, 163, 185, 215, 247, 253, and 286 being described earlier [16], [17] as well as 354 and JH029, which were obtained using the same methodology. Importantly, for *in vitro* experiments all cells were freshly isolated from early passage xenografts and cultured in low passages as adherent cells or anchorage-independent spheres. Spheres are enriched in CD133^+^ cells (**Fig. S1A**) and several other markers that have been associated with a CSC phenotype as compared to adherent cells [18].

### Metformin decreases the expression of CSCs markers

**Figure 1:**
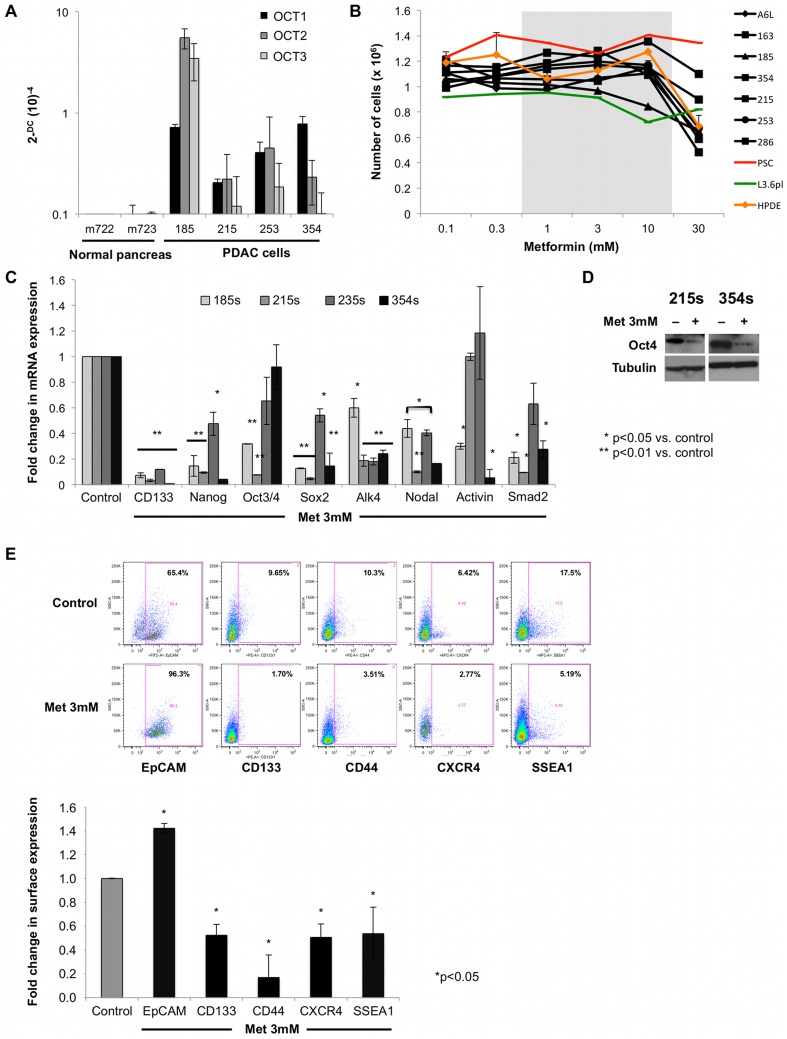
Metformin targets pancreatic cancer stem cells. (**A**) Primary PDAC cells, but not normal pancreas cells express organic cation transporter 1, 2, and 3 (n = 3). (**B**) Definition of the therapeutic range for metformin in primary PDAC cells. Number of cells grown in the presence of the indicated concentrations of metformin for 24 h (n = 6). (**C**) qPCR analysis of CSCs-associated genes in spheres treated with 3 mM of metformin for 7 days. Data are normalized to the housekeeping gene and are presented as fold change in gene expression relative to control cells (n = 6). (**D**) Representative Western blot illustrating reduced Oct4 protein expression in response to metformin treatment (n = 3). (**E**) Representative flow cytometry analysis for CSCs markers in spheres treated for 7 days with 3 mM of metformin as compared to untreated spheres (**upper panel**). Summary of data for PDAC-185, A6L, 215, 253, and 354 is shown (**lower panel**; n = 6).

First, we established the expression of the organic cation transporter 1, 2, and 3 (OCT1-3) in our primary PDAC cells (**Fig. 1A**), which are crucial for the cellular uptake of metformin. Metformin was used at concentrations that are not directly toxic to primary PDAC cells and non-transformed pancreatic cells (PSC, pancreatic stellate cells; HDPE, human ductal pancreatic epithelial cells) (**Fig. 1B**), which are significantly lower as compared to concentrations used in previous studies with PDAC cell lines (10–30 mM) [14]. Next, we found significant changes in mRNA levels of CSCs genes (CD133, Alk4, Nodal, Activin and Smad2) and pluripotency-associated genes (Nanog, Oct4 and Sox2) following treatment with metformin (**Fig. 1C**; utilized primers are listed in Table S1), which were also confirmed at the protein level (**Fig. 1D**). Strikingly, metformin appeared to preferentially eliminate CSCs as CSC-marker positive cells CD133, CD44, CXCR4 and SSEA-1 declined, while the epithelial differentiation maker EpCAM increased (**Fig. 1E**).

### Metformin selectively diminishes in vitro and in vivo tumorigenicity

**Figure 2:**
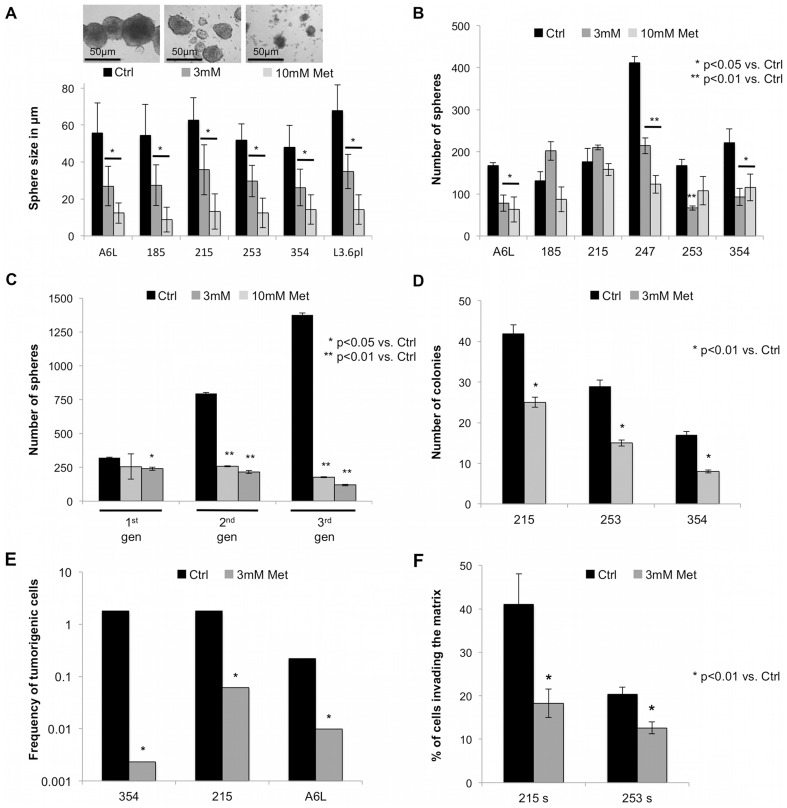
Metformin diminishes *in vitro* and *in vivo* tumorigenicity. (**A**) Metformin decreases the size of spheres. Representative images of spheres obtained after treatment with metformin for 7 days. Quantification of sphere size (n≥6). (**B**) Sphere formation capacity in the presence or absence of metformin for 7 days (n≥6). (**C**) Self-renewal capacity of cancer stem cells isolated from tumors responding poorly in terms of first passage sphere forming capacity. Cells were continuously passage as secondary and tertiary spheres treated with metformin or vehicle only during first generation sphere formation (n = 6). (**D**) Colony formation for PDAC-185, A6L, 215, 253, and 354 evidenced by 0.05% crystal violet after 21 days (n = 3). (**E**) Rarefication of *in vivo* tumorigenic cancer stem cells in spheres treated with metformin as compared to vehicle. (**F**) Invasion of sphere-derived cells after 24 h of treatment with metformin or control (n = 3).

We next examined the functional effects of metformin on the self-renewal capacity of CSCs. The sphere formation assay showed a strong decrease in the size of formed spheres by metformin (**Fig. 2A** **& S1B**) via inhibition of the expansion of the progenies of CSCs, which represent the bulk of the cells of the formed spheres. Even more importantly, we found that metformin significantly decreased the number of actually formed spheres with the same efficiency at 3 and 10 mM (**Fig. 2B**). In order to examine the long-term effect of metformin treatment on the self-renewal capacity of cells that did not show significant difference during first passage sphere formation (Panc-185 and Panc-215), secondary and tertiary spheres were initiated without further metformin treatment. Formation of spheres in the second and third passages was drastically hampered suggesting that metformin treatment had irreversibly eliminated the majority of CSCs by inhibition or abrogation of their self-renewal capacity (**Fig. 2C**). Consistently, pre-treatment with metformin resulted in the formation of fewer and smaller colonies as compared to control (**Fig. 2D**). The gold standard for CSC activity represents *in vivo* tumorigenicity. The frequency of tumorigenic cells derived from PDAC-354, 215, and A6L spheres was regularly very high with values below 1∶500 and became markedly rarified by metformin treatment (**Fig. 2E**). Finally, we found that metformin pretreatment of pancreatic CSCs subsequently reduced their migratory (**Fig. S2A**) and invasive capacity (**Fig. 2F**).

### Metformin specifically eliminates pancreatic CSCs

**Figure 3:**
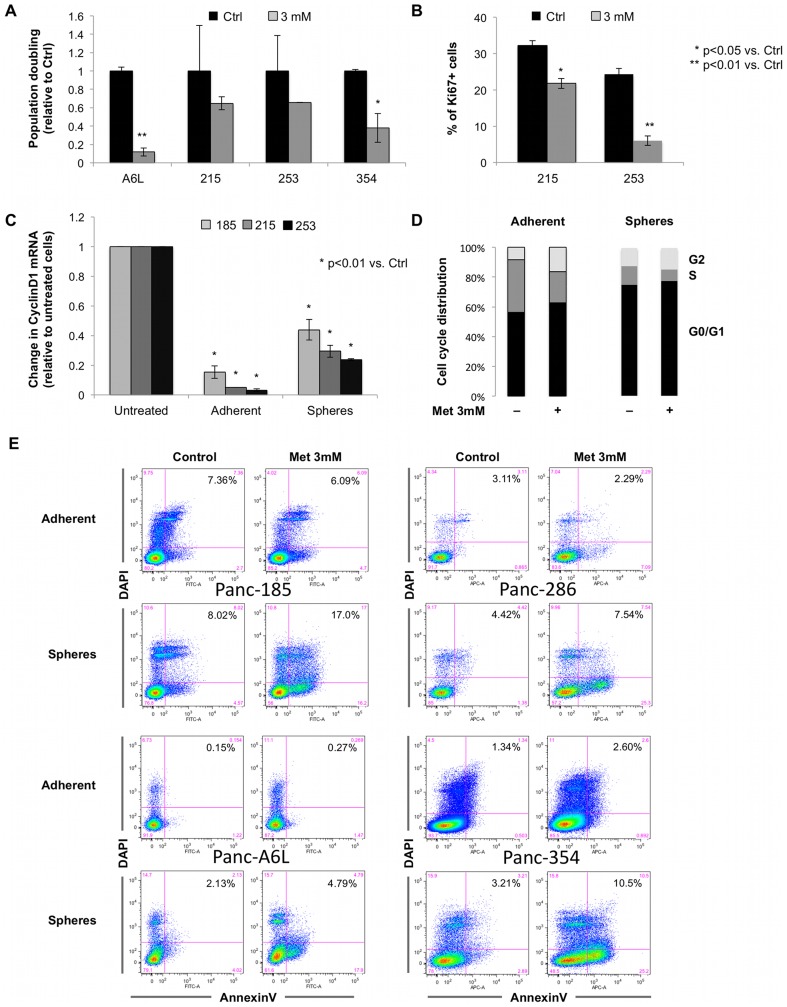
Metformin specifically eliminates cancer stem cells. (**A**) Number of cells grown in the presence of the indicated concentrations of metformin for 24 h. (n = 3). (**B**) Quantification for Ki67 and DAPI in adherent cells after 7 d of treatment with metformin or control (n = 3). (**C**) qPCR analysis for CyclinD1 in adherent and sphere-derived cells after 7 d of treatment with metformin or control. Data are normalized to the housekeeping gene and are presented as fold change in gene expression relative to untreated cells (n = 6). (**D**) Cell cycle analysis determined by Propidium Iodide staining in adherent cells and spheres after 7 d of treatment with metformin or control (n = 3). (**E**) Cytometry analysis of apoptotic cells by double staining for Annexin V/DAPI after treatment with metformin or control for adherent versus sphere-derived cells (n = 3).

Population doubling of adherent cells was markedly reduced by metformin with strong intertumoral variation (**Fig. 3A**). Consistently, Ki67 expression was diminished suggesting inhibition of cell proliferation (**Fig. 3B**), which was confirmed by reduced expression of cyclinD1 at mRNA (**Fig. 3C**) and protein level (**Fig. S2B**). Next, we analyzed the cell-cycle profile of adherent cells versus CSCs by flow cytometry. Interestingly, the data showed that the functional effect of metformin on cell cycle progression was much more marginal for sphere-derived cells suggesting a distinct mechanism responsible for their subsequent loss during extended treatment (**Fig. 3D**). Indeed, while metformin did not significantly alter the rate of apoptotic adherent cells as evidenced by AnnexinV/Dapi staining (1.23±0.37 fold change), it resulted in a strong induction of apoptosis in sphere-derived cells (2.95±1.10 fold change; P<0.01) (**Fig. 3E**). These data are in line with a preferential elimination of CSCs by metformin, whereas its effects on differentiated progeny are mostly related to inhibition of proliferation.

### Mechanism of action

**Figure 4:**
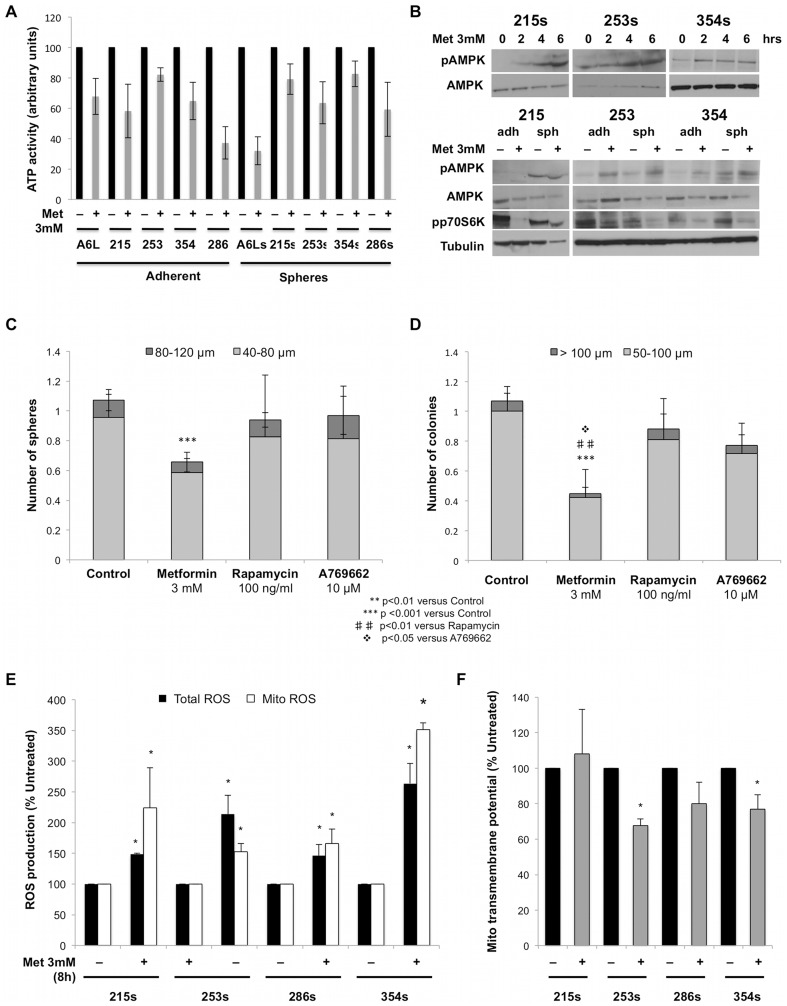
Mechanism of action. (**A**) Cellular ATP levels in adherent cells and spheres after 7 d of treatment with metformin or control (n = 3). (**B**) ***Upper panel***: Western blot analysis of pAMPK, AMPK, and GAPDH in spheres treated with metformin or control. ***Lower panel***: Western blot analysis of pAMPK, pp70S6K, and GAPDH in adherent cells and spheres treated with metformin or control for 7 days (n = 3). (**C**) Overall effect of metformin (3 mM), inhibition of mTOR (rapamycin; 100 ng/ml), and direction activation of AMPK (A769662; 10 µM) on sphere formation and (n = 6). (**D**) colony formation for PDAC-215, 253, and 354 cells (n = 3). (**E**) Total and mitochondrial (Mito) ROS production after 8 hours of control or metformin treatment. (**F**) Mitochondrial transmembrane potential after 8 hours of control or metformin treatment.

Metformin uniformly reduced ATP level both in adherent cells and spheres across the panel of investigated tumors including tardy responders (**Fig. 4A**). Consistently, metformin induced a time-dependent increase of pAMPK in CSCs (**Fig. 4B** **, upper panel**) with no apparent difference to non-CSC (data not shown). Moreover, we observed a similar net decrease in p70S6K, suggesting an efficient blockade of the mTOR pathway (**Fig. 4B** **, lower panel**). As the mTOR pathway is a central regulator of autophagy, we analyzed whether metformin might induce characteristic hallmarks of autophagy in CSCs versus non-CSCs, but our results do not support the notion that the selective elimination of CSCs by metformin can be rationalized by alterations in autophagy (**Fig. S3A/B**). To provide further evidence that AMPK/mTOR pathway is not mediating the deleterious effects of metformin on CSCs, we investigated if the effects of metformin are mimicked by the direct AMPK activator A769662 and/or the mTOR inhibitor rapamycin (Rapa). Strikingly, neither sphere formation (**Fig. 4C**) nor colony formation (**Fig. 4D**) was significantly inhibited by the two inhibitors, essentially ruling out AMPK/mTOR as the driving mechanism for metformin in targeting pancreatic CSCs.

Next, we hypothesized that the particular sensitivity of CSCs to metformin might be attributable to anti-oxidant properties to metformin [19]. Indeed, metformin treatment significantly increased mitochondrial production of reactive oxygen species (ROS) in CSCs derived from all the used tumors, but these changes were more pronounced in rapid responders (PDAC-253 and 354) as compared to the tardy responders PDAC-215 and 286 (where metformin effects are not detectable in first generation sphere formation) (**Fig. 4E**). Interestingly, consistent data were obtained for the mitochondrial transmembrane potential, which was most prominently reduced in rapid responders (**Fig. 4F**). Using the mitochondrial probe TMRE, we demonstrate that in these tumors metformin decreased their mitochondrial transmembrane potential (used as a general marker of mitochondrial toxicity and induction of apoptosis), while it remained unchanged or only slightly reduced in tardy responders. This direct effect on mitochondria may also explain the differential sensitivity between non-CSCs and CSCs as the latter appear to be more dependent on their mitochondria for generating energy, while non-CSCs mainly rely mitochondria-independent sources such as aerobic glycolysis (Warburg effect) [20]. In addition, we also confirmed that the effect of metformin on mitochondria was independent of AMPK/mTOR as neither the direct AMP activator A769662 nor the mTOR inhibitor rapamycin were capable of mimicking the effects of metformin (**Fig. S4A/B**).

### Metformin stalls PDAC progression in vivo

**Figure 5:**
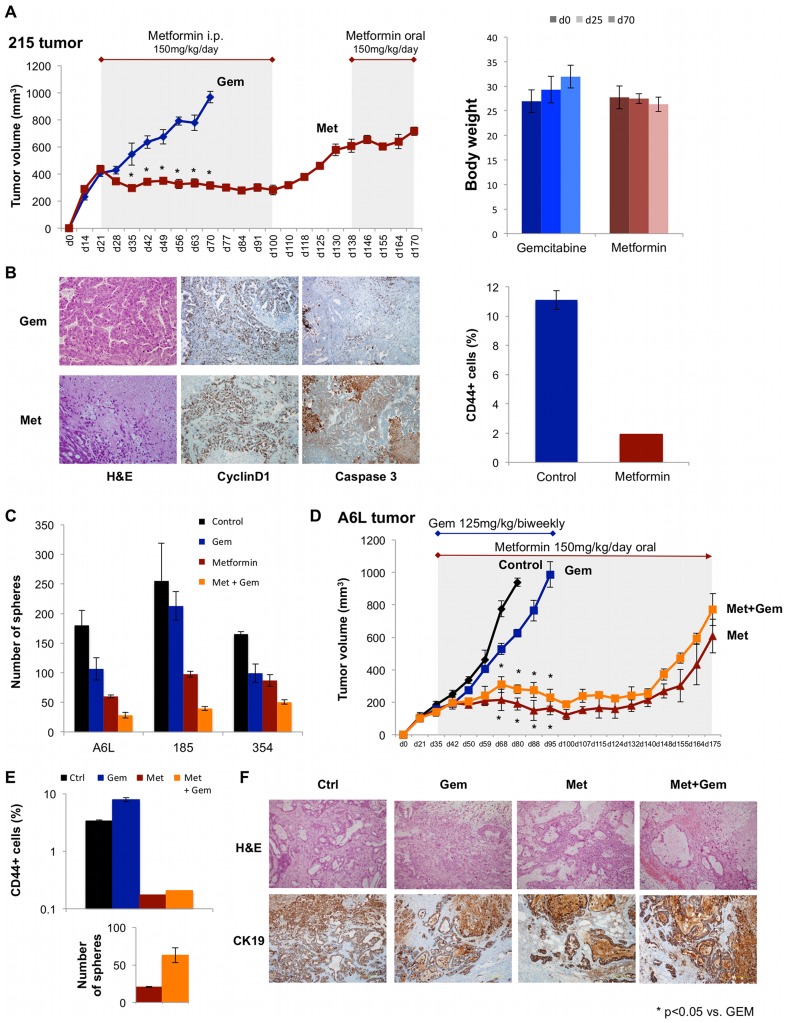
Metformin stalls PDAC progression in vivo. (**A**) ***Left panel***: PDAC-215 tissue was implanted and treatment was allocated after initial tumor take was verified. Mice were treated with either gemcitabine (Gem) or metformin (Met). Metformin was discontinued on d100 to test the potential of the remaining lesions to initiate disease relapse. After documented disease relapse, metformin treatment was re-administered. ***Right panel***: Body weight during treatment. (**B**) ***Left panel***: Histological analysis: Hematoxylin & Eosin (H&E), CyclinD1, and Caspase3. ***Right panel***: Content for CD44+ cells. (**C**) Effects of *in vitro* treatment as indicated on sphere formation capacity. (**D**) PDAC-A6L tissue implanted in mice and treated as indicated. (**E**) Content for CD44+ cells (***upper panel***) and sphere formation capacity (***lower panel***). (**F**) Histological analysis: H&E and cytokeratin19 (CK19; n = 6).

First, we studied the effects of metformin *in vivo* using a brief 7-day metformin treatment of PDAC-185. During the subsequent follow-up of 40 days we observed a significant reduction in tumor growth and a complete remission in 3 out 8 tumors for metformin treated-mice as opposed to no remission in the control group (**Fig. S5**). Consistent data were obtained for other PDACs with metformin monotherapy being highly effective in inducing disease regression (**Fig. 5A–F**). Several important observations were made during these studies. First, no gross toxicity was observed during prolonged metformin treatment as body weight remained unchanged (**Fig. 5A** **, right panel**). Secondly, tumors slowly, but regularly relapsed after withdrawal of metformin on day 100 (**Fig. 5A** **, left panel**). Thirdly, while the addition of gemcitabine *in vitro* showed an additive effect on the CSC population (**Fig. 5C**), no additive effect could be noted *in vivo* for metformin plus gemcitabine (**Fig. 5D**).

**Figure 6:**
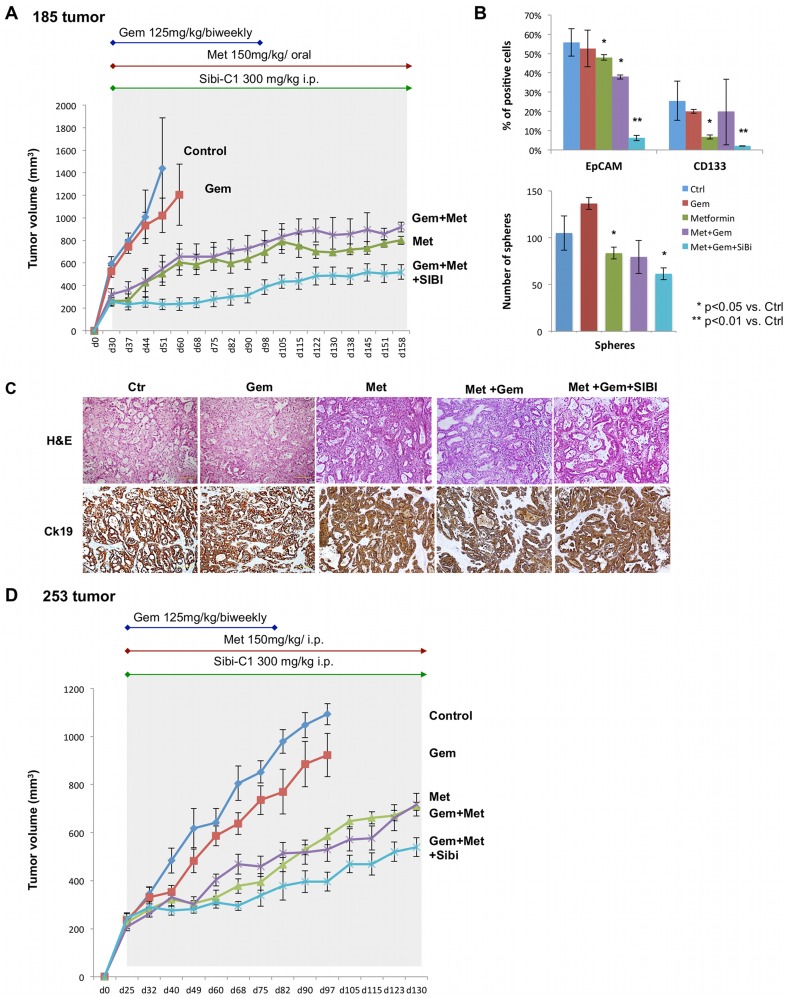
Additional targeting of the stroma prevents tumor relapse. (**A**) PDAC-185 tissue implanted in mice and treated as indicated including the smoothened inhibitor SIBI-C1. (**B**) Content for EpCAM+ and CD133+ cells, respectively, (***upper panel***) and sphere formation capacity (***lower panel***). (**C**) Histological analysis: Hematoxylin&Eosin (H&E) and cytokeratin19 (CK19). (**D**) mTOR inhibitor resistant PDAC-253 tissue implanted in mice and treated as indicated (n = 6).

These data prompted us to hypothesize that gemcitabine was not appropriately delivered to the PDAC tissue [21] and/or the stroma protected the CSCs from the deleterious effects of metformin by promoting their stemness and subsequently resistance [22]. Therefore, we added the smoothened inhibitor SIBI-C1 as a stroma/stellate cells-targeting agent to the combination of gemcitabine plus metformin. Since we have previously shown that the combination of gemcitabine plus smoothened inhibitor does not prevent relapse [10] and gemcitabine currently represents standard treatment for PDAC, we only tested this combination. In addition to enhancing tissue delivery of gemcitabine,[21] the triple combination also resulted in a doubling of tissue metformin concentration (23.1±0.82 versus 10.8±1.9 mg/g). Subsequently reproducible disease regression was observed (**Fig. 6A–C**) and, even more importantly, this combination was also effective in tumors previously shown to be resistant to mTOR inhibition (**Fig. 6D**) [23].

## Discussion

Here we demonstrate that the heterogeneous populations of cancer cells harbored in primary human PDAC tissues differ in their response to metformin depending on their level of stemness. While the bulk of more differentiated cancer cells reacted to treatment with metformin with cell cycle arrest, a subset of cells with distinct stemness features, namely CSCs actually underwent rapid apoptotic death due to energy crisis. Although the most apparent effect for metformin treatment in primary spheres was a marked reduction in sphere size consistent with a reduced proliferation rate of the more differentiated cells harbored in these spheres, subsequent serial passaging of the spheres clearly revealed their almost complete and irreversible loss of clonogenic propagation ability, despite the fact that metformin treatment was stopped already after the first passage. These data demonstrate that metformin virtually exhausted the CSC fraction, but are also consistent with the notion that non-CSC do not replenish the pancreatic CSC pool after termination of metformin treatment. Consistently, transplantation of the pretreated cells into immunocompromised mice revealed that the tumorigenicity of the cells was indeed drastically reduced by short-term exposure to metformin. Most importantly, however, *in vivo* treatment of established primary human cancers strongly responded to metformin with disease regression and subsequent stable disease.

The identified mechanism of action for metformin in CSCs has remained largely unknown. Most investigations in bulk tumor cells support a simplified working model in which metformin exerts anti-tumoral effects by indirectly activating AMPK followed by subsequent suppression of mTOR [8]. Directly targeting mTOR with rapalogs has also been considered as a target for anticancer drug development. While this was effective in patients with several cancers [24], preclinical studies in PDAC did not suggest a particular high response rate (23%) [23]. Importantly, one of the insensitive tumors in that study was PDAC-215, which we were able to also test in the present study using metformin. CSCs derived from this tumor showed a robust inhibition of secondary sphere formation *in vitro* and efficient tumor regression *in vivo*. On the other hand, direct activation of mTOR by A769662 or inhibition of mTOR by rapamycin did not mimic the strong effects that we achieved with metformin suggesting that treatment effects of metformin in pancreatic CSCs are independent of alterations of the AMPK/mTOR axis.

Human PDAC cells are known for their inherent tolerance to nutrition starvation, which enables them to survive under a hypovascular (austerity) tumor microenvironment. It is an emerging paradigm that normal stem cells are characterized by predominant aerobic glycolysis and suppressed mitochondrial oxidation with subsequently lower mitochondrial ATP production and ROS release [25]. Our data are in line with the notion that pancreatic CSCs actually bear a highly mitochondrial-dependent metabolic profile, which is in striking contrast to normal stem cells, but also distinguishes them from the bulk cancer cells. It has previously been shown that metformin is slowly accumulated 1000-fold within mitochondria and directly inhibits Complex 1 (NADH dehydrogenase), which translocates four protons from the mitochondrial matrix to the intermembrane space, thus producing a proton gradient. The electron transport chain and oxidative phosphorylation are coupled by this proton gradient across the inner mitochondrial membrane, and their inhibition appears to be particularly lethal for CSCs [26]. Therefore, drugs such as metformin that target the oxidative mitochondrial metabolism represent powerful therapeutic tools for attacking the CSC pool.

The ability of metformin to selectively eliminate CSCs is in stark contrast to the effects of gemcitabine, a chemotherapeutic drug that kills bulk cancer cells, but not cancer stem cells [15]. Based on their distinct properties, metformin should synergize with gemcitabine to reduce both non-CSCs and CSCs in the mixed population. Indeed, it was only recently shown in four genetically different types of breast cancer cell lines that metformin selectively kills CSCs and that the combination of metformin and the standard chemotherapeutic agent doxorubicin killed both CSCs and non-CSCs *in vitro* as well as reduced tumor mass and prolonged remission more effectively *in vivo* than either drug alone [27]. While we could confirm this hypothesis for PDAC *in vitro*, our *in vivo* treatment studies suggest that gemcitabine is actually dispensable as long as metformin is continued throughout the study. While these data certainly warrant further validation it appears reasonable to speculate that metformin monotherapy could represent a novel treatment option for patients that are too fragile to tolerate the side effects of the chemotherapeutic agent gemcitabine [28].

Unfortunately, however, all tested tumors eventually progressed under metformin therapy. It remains to be determined if CSCs exposed to long-term metformin treatment switch their metabolic phenotype rendering them resistant to metformin's effects on mitochondria (acquired resistance) or whether a preexisting subpopulation of CSCs is actually resistant to metformin and eventually becomes the dominating clone (inherited resistance). This issue should be addressed in future studies by comparing CSCs isolated from tumors that regressed under metformin treatment and CSCs isolated from tumors that eventually progressed under the same conditions. Extensive characterization of resistant clones should provide crucial insights as to whether this is actually preventable or second line treatment options could be offered.

Importantly, however, the addition of a stroma targeting smoothened inhibitor appears mandatory to achieve stronger and longer lasting response rates. The most likely explanation could be two fold. One contributing factor to the failure of systemic therapies may be the abundant tumor stromal content that is the characteristic of PDAC. The stroma represents the majority of the tumor mass, and consists of a dynamic assortment of extracellular matrix components and non-neoplastic cells including fibroblastic, vascular, and immune cells. Recent work has revealed that the stroma supports CSCs, [18], [22], [29] promotes invasiveness and metastasis as well as simultaneously serves as a physical barrier to drug delivery [21]. Therefore, hedgehog pathway inhibition, in addition to providing better access for systemically administered drug, may also abrogate the CSC niche rendering them more susceptible to the treatment effects of metformin and/or gemcitabine. Future more extensive studies will have to show if the development of resistance can be efficiently prevented by this treatment strategy.

It may be argued that the metformin concentrations used in our *in vitro* studies, albeit already significantly lower than those utilized in previous studies, are still non-achievable *in vivo* and therefore irrelevant from a mechanistic point of view. However, it is important to note that metformin accumulates in tissues and particularly in mitochondria at concentrations several-fold higher than those measured in blood [26]. This is particularly true for cells that are equipped with the respective transporters relevant for metformin uptake, namely OCT1-3. As we show here, PDAC cells express all three isoforms, but show particularly high expression of OCT1. While it is difficult to assess the true intracellular metformin concentrations in PDAC cells due to the massive stroma content and necrotic areas in harvested tumors, our analysis of the metformin concentration revealed higher metformin concentrations in the PDAC tissue (10.8 mg/g of tissue) than in the liver (8.9 mg/g) and even more so if metformin was administered in combination with the smoothened inhibitor SIBI-C1 (23.1 mg/g). Therefore, our mechanistic *in vitro* studies are indeed highly relevant for the *in vivo* setting.

Metformin bears an exceptional safety profile as only hepatocytes, but not other non-transformed (stem) cells express OCT for efficient cellular uptake of metformin. Several clinical trials (NCT01210911, NCT01167738, and NCT01488552) are currently testing metformin in locally advanced and metastatic PDAC. This will hopefully provide a definitive assessment of the clinical effects of metformin in PDAC. It would be of particularly interest whether patients will also ultimately progress during metformin treatment and whether this could be assessed and/or predicted by the analysis of circulating cancer (stem) cells. Their prospective isolation during relapse may provide important mechanistic insights into the cause of relapse.

## Materials and Methods

### Tumor samples

After written informed consent had been obtained from the patients, excess tissues from resected pancreatic carcinomas was xenografted at Johns Hopkins Medical Institutions (Ethics board approval: JHMIRB: 05-04-14-02 “A Feasibility Study for Individualized Treatment of Patients with Advanced Pancreatic Cancer”) and Hospital de Madrid - Centro Integral Oncológico Clara Campal (FHM.06.10 “Establishment of bank for tumors and healthy tissue in patients with cancer”), respectively, under the indicated Institutional Review Board-approved protocols [30]. Briefly, excess tumor tissues not needed for clinical diagnosis during routine Whipple resections performed by surgeons that were not involved in the present study were subsequently implanted into immunocompromised mice. All patient information was made anonymous by removal of any information, which identifies, or could lead to the identification of the patient. None of the patients had undergone neoadjuvant radiation or chemotherapy prior to resection of the tumor.

### Primary human PDAC cells

For in vitro studies, tissue fragments were minced, enzymatically digested with collagenase (Stem Cell Technologies, Vancouver, British Colombia) for 90 min at 37°C [10] and after centrifugation for 5 min at 1,200 rpm the pellets were resuspended and cultured in RPMI, 10% FBS and 50units/ml penicillin/streptomycin. For some experiments, the human PDAC cell line L3.6pl was used as previously described [15].

### Cancer stem cell-enriching culture

PDAC spheres were generated and expanded in DMEM-F12 (Invitrogen, Karlsruhe, Germany) supplemented with B-27 (Gibco, Karlsruhe, Germany) and bFGF (PeproTech EC, London, United Kingdom). 10^3^ cells/ml were seeded in ultra-low attachment plates (Corning B.V., Schiphol-Rijk, The Netherlands) as described previously.[31] After 7 d of incubation, spheres were typically >75 µm large with ∼97% CD133high. For serial passaging, 7-day-old spheres were harvested using 40 µm cell strainers, dissociated to single cells with trypsin, and then re-grown for 7 d. Cultures were kept no longer than 4 weeks after recovery from frozen stocks (passage 3–4).

### In vivo treatment of established PDACs

All animal experiments were conducted in accordance with institutional guidelines and were approved by the Institutional Animal Care and Use Committee of the CNIO (Protocol PA34/2012 – “Xenotransplant model for human pancreatic cancer”). Animals were housed and maintained in laminar flow cabinets under specific pathogen-free conditions. Briefly, 8 mm^3^ pieces of primary, *in vivo* expanded pancreatic cancer tissue pieces were either orthotopically or subcutaneously implanted into the pancreas of 6–8 weeks old female nude mice (Harlan Europe) as described previously [10], [30], [32]. Tumor size and body weights of all animals were measured weekly. Size of the subcutaneous tumors was measured by caliper and calculated as length×width×depth. Mice were randomized to the respective treatment groups after documented tumor take (200+mm^3^). Size and weight of the pancreatic tumors were monitored. Gemcitabine was administered biweekly (125 mg/kg i.p.; Lilly, Indianapolis, Indiana). Metformin was administered daily via i.p. injections (150 mg/kg BW; Sigma-Aldrich) or the drinking water (150 mg/kg BW). SIBI-C1 was administered twice daily via i.p. injection (300 mg/kg BW; Siena Biotech, Siena, Italy).

#### Statistical analyses

Results for continuous variables are presented as means ± standard deviation (SD) unless stated otherwise. Treatment groups were compared with the independent samples t test. Pair-wise multiple comparisons were performed with the one-way ANOVA (two-sided) with Bonferroni adjustment. P values<0.05 were considered statistically significant. All analyses were performed using SPSS 17.0 (SPSS Inc., Chicago, Illinois).

More information can be found as **Materials and Methods S1**.

## Supporting Information

Figure S1 **(related to** **Figure 1** **&** **2** **) Metformin targets pancreatic cancer stem cells.** (**A**) Adherent and sphere-derived cells isolated from different PDAC tissues were treated for 7 days with metformin or control and analyzed for CD133 expression by flow cytometry (gates were set according to the respective isotype control). (**B**) Sphere formation capacity after treatment for 7 days with metformin or control. Representative images for the respective PDACs are shown (n≥3).
(TIF)

Figure S2 **(related to** **Figure 3** **) Metformin specifically eliminates cancer stem cells.** (**A**) Quantification of scrape-induced migration after 24 h of treatment with metformin or control (n = 3). (**B**) Western blot analysis for cyclinD1 expression in adherent and spheres treated with 3 mM of metformin for 7 days. Quantification is shown relative to the housekeeping gene GAPDH and normalized to control (n≥3).
(TIF)

Figure S3 **(related to** **Figure 4** **) Role of autophagy.** (**A**) qPCR analysis of ATG12 in adherent and spheres treated with 3 mM of metformin for 7 days. Data are normalized to the housekeeping gene. ATG12 as a marker for autophagy was not consistently altered by metformin in the different tumors and did not show distinct alterations between CSCs versus non-CSCs. (**B**) Western blot analysis for LC3 expression in adherent and spheres treated with 3 mM of metformin for 7 days. Also on the protein level, only slightly increased LC3b expression was detected after the treatment with metformin both in spheres and adherent cells as well as in tumors xenograft treated with metformin *in vivo* (n≥3).
(TIF)

Figure S4 **(related to** **Figure 4** **) Role of AMPK/mTOR.** (**A**) Mitochondrial ROS production after 8 hours of treatment with metformin (Met 3 mM), AMPK activator A769662 (10 µM), or rapamycin (Rapa 10 ng/ml). (**B**) Mitochondrial transmembrane potential after 8 hours of indicated treatment (n≥3).
(TIF)

Figure S5 **(related to** **Figure 5** **)** ***In vivo*** **targeting of pancreatic cancer stem cells.** (**A**) PDAC-185 cells were implanted into immunocompromised mice and treatment was allocated on d7 after initial tumor take was verified. Mice were treated with metformin alone until d14. During the subsequent follow-up we observed a significant reduction in tumor growth and a complete remission in 3 out 8 tumors for metformin treated-mice as opposed to no remission in the control group (n = 6). (**B**) Subsequent immune-histochemistry analysis on day 40 and therefore 26 days after termination of metformin treatment, revealed an increase in necrotic areas of tumors treated with metformin and a consistent decrease in expression of CyclinD1 and pp7056K. (**C**) qPCR analysis for stem cell genes on day 40 and therefore 26 days after termination of metformin treatment.
(TIF)

Table S1 **List of utilized primers.**
(TIF)

Materials and Methods S1 **Western Blot Analysis, Measurement of intracellular ATP levels, RNA preparation and RT-PCR, Flow cytometry, Invasion and migration assay, Clonogenic assay, Apoptosis assay, Immunohistochemistry, Cell-cycle analysis.**
(PDF)
