# Supplementary figures and images for: Metformin Targets the Metabolic Achilles Heel of Human Pancreatic Cancer Stem Cells
Source: PLoS One. 2013 Oct 18;8(10):e76518. doi: 10.1371/journal.pone.0076518 (PMC3799760; doi:10.1371/journal.pone.0076518)

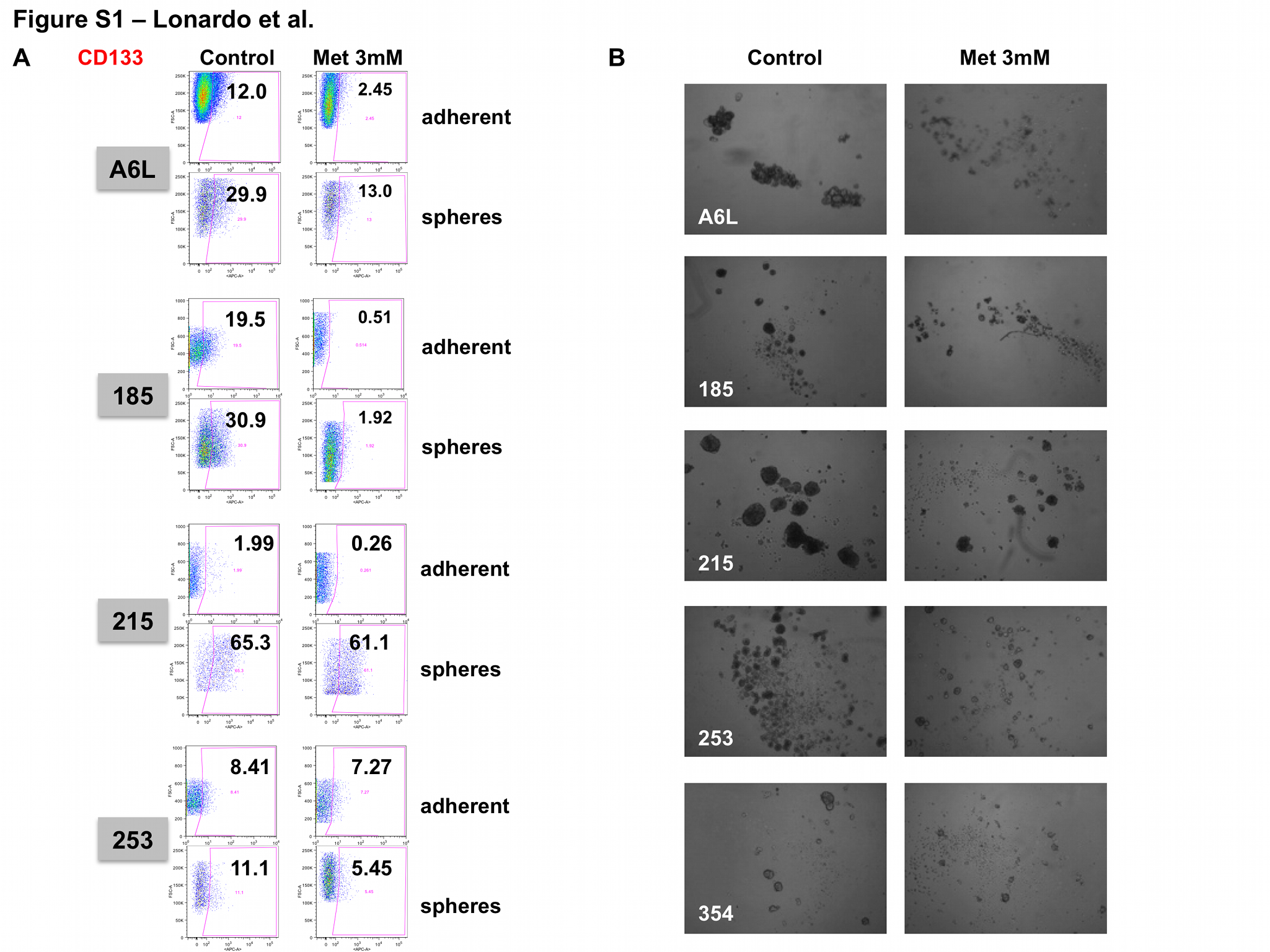

Supplement: Figure S1 — (related to Figure 1 & 2 ) Metformin targets pancreatic cancer stem cells. (A) Adherent and sphere-derived cells isolated from different PDAC tissues were treated for 7 days with metformin or control and analyzed for CD133 expression by flow cytometry (gates were set according to the respective isotype control). (B) Sphere formation capacity after treatment for 7 days with metformin or control. Representative images for the respective PDACs are shown (n≥3). (TIF) [file pone.0076518.s001.tif]

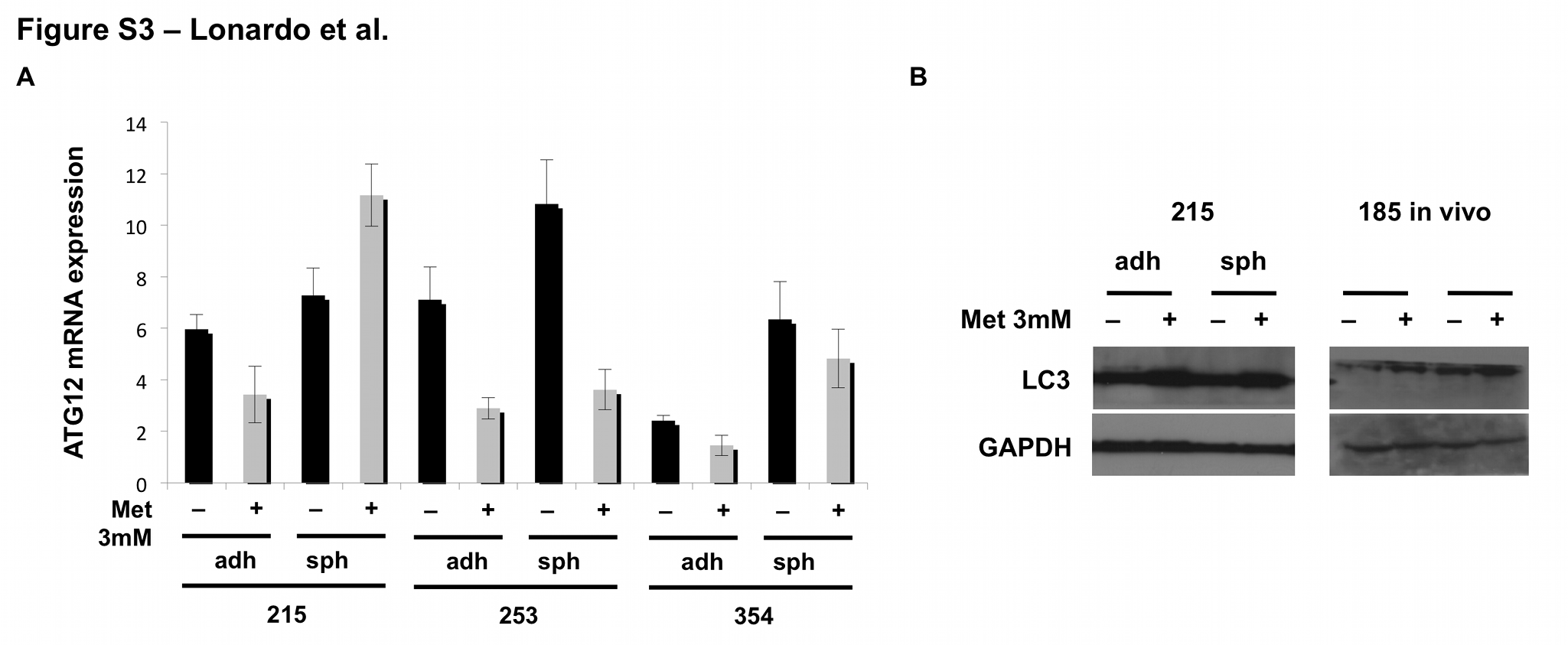

Supplement: Figure S3 — (related to Figure 4 ) Role of autophagy. (A) qPCR analysis of ATG12 in adherent and spheres treated with 3 mM of metformin for 7 days. Data are normalized to the housekeeping gene. ATG12 as a marker for autophagy was not consistently altered by metformin in the different tumors and did not show distinct alterations between CSCs versus non-CSCs. (B) Western blot analysis for LC3 expression in adherent and spheres treated with 3 mM of metformin for 7 days. Also on the protein level, only slightly increased LC3b expression was detected after the treatment with metformin both in spheres and adherent cells as well as in tumors xenograft treated with metformin in vivo (n≥3). (TIF) [file pone.0076518.s003.tif]

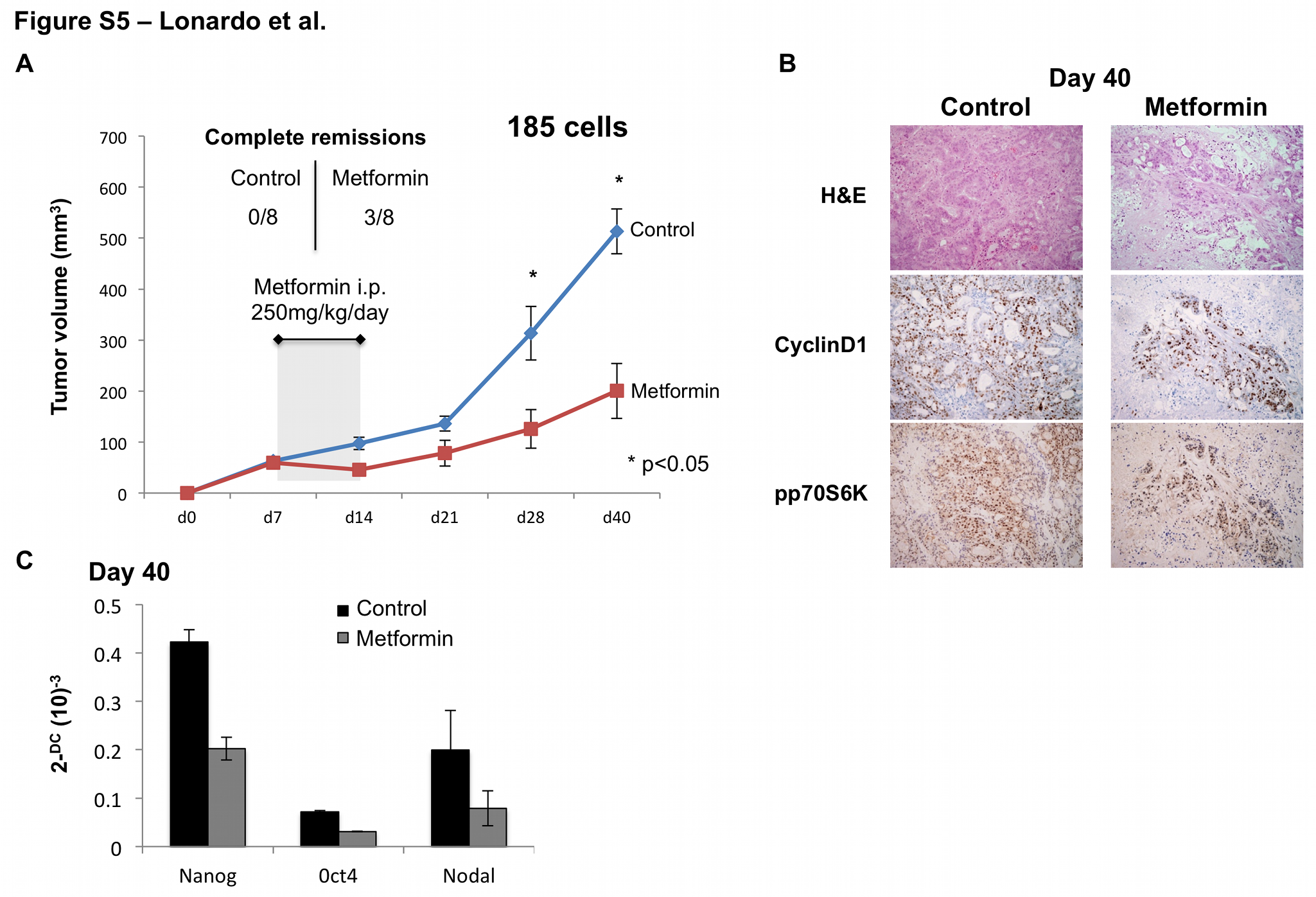

Supplement: Figure S5 — (related to Figure 5 ) In vivo targeting of pancreatic cancer stem cells. (A) PDAC-185 cells were implanted into immunocompromised mice and treatment was allocated on d7 after initial tumor take was verified. Mice were treated with metformin alone until d14. During the subsequent follow-up we observed a significant reduction in tumor growth and a complete remission in 3 out 8 tumors for metformin treated-mice as opposed to no remission in the control group (n = 6). (B) Subsequent immune-histochemistry analysis on day 40 and therefore 26 days after termination of metformin treatment, revealed an increase in necrotic areas of tumors treated with metformin and a consistent decrease in expression of CyclinD1 and pp7056K. (C) qPCR analysis for stem cell genes on day 40 and therefore 26 days after termination of metformin treatment. (TIF) [file pone.0076518.s005.tif]
