# Supplementary material for: Metformin Targets the Metabolic Achilles Heel of Human Pancreatic Cancer Stem Cells
Source: PLoS One. 2013 Oct 18;8(10):e76518. doi: 10.1371/journal.pone.0076518 (PMC3799760; doi:10.1371/journal.pone.0076518)
Supplement: Materials and Methods S1 — Western Blot Analysis, Measurement of intracellular ATP levels, RNA preparation and RT-PCR, Flow cytometry, Invasion and migration assay, Clonogenic assay, Apoptosis assay, Immunohistochemistry, Cell-cycle analysis. (PDF) [file pone.0076518.s007.pdf]

Supplemental Material & Methods

Western Blot Analysis. PVDF membranes containing electrophoretically separated proteins from human primary PDAC cells and spheres were probed with antibodies against pAMPK (thr172) (2535), AMPK (2532), pp70S6K (thr389) (9205), Cyclin D1 (29265), Oct4a (2890), ß-tubulin (2146; all Cell Signaling) and GAPDH, (ab82633; Abcam, Cambridge, United Kingdom) treated with peroxidase-conjugated sheep anti-mouse or rabbit Ig secondary antibody and then visualized by enhanced chemoluminiscence (both from GE Healthcare, Buckinghamshire, United Kingdom).

Measurement of intracellular ATP levels. Intracellular ATP was measured by a luciferin/ luciferase-based assay. Cells were grown under each experimental condition for indicated times, harvested, and counted. Aliquots containing equal number of cells were processed following manufacturer's guidelines (Roche, Penzberg, Germany).

RNA preparation and RT-PCR. Total RNAs from human primary PDAC cells and spheres were extracted with TRIzol kit (Life Technologies, Carlsbad, California) according to the manufacturer’s instructions. One microgram of total RNA was used for cDNA synthesis with SuperScript II reverse transcriptase (Life Technologies) and random hexamers. Quantitative real-time PCR was performed using SYBR Green PCR master mix (Qiagen, Hilden, Germany), according to the manufacturer’s instructions. The list of utilized primers is depicted in **Table S1**.

Flow cytometry. To identify pancreatic CSCs, the following antibodies were used: anti-CD133/1-APC or PE; (Miltenyi, Bergisch-Gladbach, Germany), anti-CXCR4-APC, SSEA-1-APC, EpCAM-FITC, CD44-PE (all from eBiosciences, San Diego, California), or appropriate isotype-matched control antibodies. DAPI was used for exclusion of dead cells (eBiosciences). All samples were analyzed by flow cytometry using a FACS Canto II (BD) and data were analyzed with FlowJo 9.2 software (Ashland, Oregon).

Invasion and migration assay. Invasion assays were performed using modified Boyden chambers filled with Matrigel™ (eBiosciences). Adherent and sphere culture derived cells were used for this experiment. Cells were added to the Matrigel™-coated inserts and 750µL of serum-free or conditioned media was added to the lower chamber. The assay chambers were incubated for 24h at 37°C. Invasive cells were fixed in 4% PFA and stained with DAPI using Prolong Gold (Invitrogen). The number of invaded cells was compared to control. For wound healing-migration assay the cells were seeded onto six-well dishes at 10^5 per well. A single scratch wound was created using a p10

pipette tip in to confluent cells. Cells were washed twice with PBS to remove cell debris, supplemented with assay medium, and monitored. Images were captured at 0, 24, 48 and 72h after wounding.

Clonogenic assay. $5x10^2$ cells were plated in six-well plate. After 7d of exposure to 3mM of metformin, 100ng/mL of rapamycin or 10µM A769662, the cells were trypsinized, and 1,000 single viable cells were plated in 100-mm Petri dishes. The cells were then incubated for 15d at 37°C. Colonies were stained with 2% crystal violet, washed with water, and counted.

Apoptosis assay. Cancer cells and CSCs were cultured in presence of 3mM of metformin for 7 days. Attached and floating cells were collected, resuspended and stained with Annexin V (550474;) after incubation with Annexin V binding buffer (556454, all from eBiosciences). Cells were then incubated with DAPI.

Immunohistochemistry. Tumor xenografts were fixed in paraffin blocks. Tissue sections were stained with: Hematoxylin and Eosin, anti-Human CyclinD1, anti-Human Caspase3, anti-Human CK19, anti-Human pp70S6K and ß-galactosidase. Color was developed using 3,3'-diaminobenzidine (0.1mg/ml, 0.02% H_2O_2, 3min) as chromogen and nuclei were stained with hematoxylin. Sections were mounted with Entellan (Merck, Darmstadt, Germany).

Cell-cycle analysis. Cells were trypsinized, washed in PBS, centrifuged, and pellets were fixed in 200µl of 70% ethanol and stored at -20°C until use. Cells were centrifuged and pellets resuspended in 200µl of PBS, and 10µg/mL of RNAse A was incubated for 1 hour at 37°C. Subsequently, cells were resuspended in propidium iodide solution (0.1% sodium citrate, 0.1% TritonX-100, and 50µg/mL propidium iodide).
